# Malignant uterine disease with concurrent myometrial contraction at MRI: a possible source of overstaging

**DOI:** 10.1590/0100-3984.2015.0057

**Published:** 2016

**Authors:** Sriluxayini Manikkavasakar, Amrutha Ramachandram, Miguel Ramalho, António P. Matos, Richard C. Semelka

**Affiliations:** 1 Department of Radiology, University of North Carolina at Chapel Hill, Chapel Hill, NC, USA.

Dear Editor,

We report a case of a 41-year-old woman with lower abdominal pain and vaginal bleeding with negative ultrasound scan except for fibroids, and elevation of serum β-hCG (244,410 mIU/mL). Differential diagnoses included ectopic pregnancy, early pregnancy failure, very early ongoing pregnancy and molar pregnancy.

**Figure 1:**
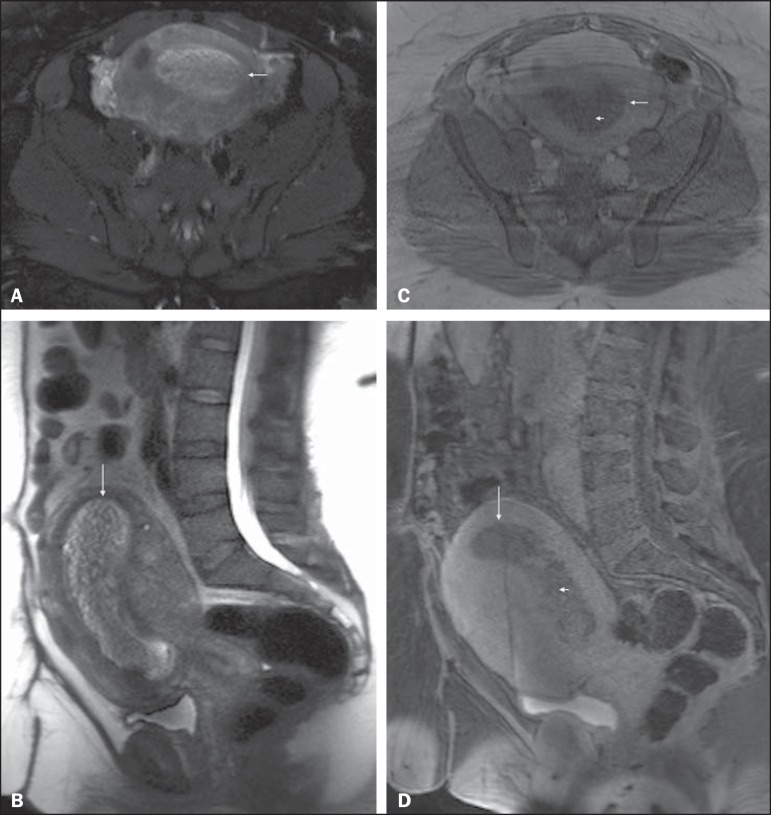
Pelvic MRI. Axial fat-suppressed (**A**) and sagittal (**B**) T2-weighted images, and post-contrast axial (**C**) and sagittal fat-suppressed (**D**) T1-weighted MRI images. An enlarged uterus is depicted, with a heterogeneous T2-weighted hyperintense lesion distending the endometrial canal (arrows, **A-D**). The lesion shows reticular enhancement on postcontrast imaging, concerning for gestational trophoblastic disease (short arrows, **C** and **D**). Note the different morphologic aspect of the anterior and posterior uterine walls between the first set of images (**A** and **B**) and those acquired later (**C** and **D**), suggesting motion in the context of contraction. These differences are more accentuated on sagittal images, showing substantial increase in thickness and bulging of the posterior myometrial wall on T2-weighted image (**B**), whereas this pattern is inverted and appearing on the anterior myometrial wall on late post-contrast T1-weighted images (**D**) (acquired with a delay of 30 minutes compared to T2-weighted images).

MRI showed an enlarged uterus with central heterogeneous T2 hyperintensity distending the endometrial canal, demonstrating reticular enhancement, concerning for gestational trophoblastic disease. There was also distortion of the junctional zone with broad intermediate-to-low signal on T2-weighted images. The variable appearance of myometrial thickness especially on postcontrast images, showing a homogeneous myometrium, facilitated the diagnosis of contractions (Figure 1). The patient underwent suction and curettage with a final diagnosis of complete hydatidiform mole (HM) (p57 negative).

Gestational trophoblastic disease (GTD) arise from placental trophoblastic tissue after abnormal fertilization and comprises a spectrum of disorders from the pre-malignant conditions of partial HM and complete HM to the malignant invasive mole, choriocarcinoma and the very rare placental site trophoblastic tumor ^(1,2)^. HM is the most common manifestation of GTD (85%) and by definition noninvasive and confined to the endometrium. A HM that invades the myometrium is termed invasive mole, and is composed of HM villi within the myometrium. Chorioadenoma destruens is a locally invasive (myometrium) manifestation of complete HM that represents 13% of cases of GTD. Two percent of complete HM cases are described as choriocarcinoma, which is locally invasive and potentially metastasizing. These three entities produce peculiarly high levels of β-hCG, while placental site trophoblastic tumor causes a rise in human placental lactogen levels, and less elevated β-hCG levels^(3,4)^. Clinical assessment is difficult early in the course of the disease, as few clinical characteristics are present to distinguish it from a normal pregnancy.

Pelvic MRI is often used as a problem-solving tool in equivocal or complicated cases of GTD, especially in the first trimester, or to assess the degree of myometrial invasion and surrounding tissues^(2,5)^. Early manifestations appear as a soft tissue cystic mass with high T2 signal intensity^(6)^. In the second trimester these lesions tend to distend the endometrium giving a "cluster of grapes appearance". Typically HMs are similar or slightly higher in T1 signal intensity than the adjacent myometrium. Contrast-enhanced MRI show areas of focal enhancement that relate to the amount of active trophoblastic tissue and also to β-hCG levels^(7)^. Marked early enhancement indicates active disease in the form of viable trophoblastic tissue.

In the setting of GTD, identification of myometrial invasion is crucial for diagnosis and staging. Uterine tumors associated with high serum β-hCG have a high incidence of myometrial contractions^(8)^. Myometrial contractions are seen as a bulge of the myometrial wall usually along with a region of low T2 signal intensity in the myometrium. They are transient and tend to disappear on subsequent data acquisitions^(9)^, as observed in our case. In the setting of endometrial tumor, radiologists should be aware of this phenomenon to avoid over-diagnosis and over-staging by misdiagnosing uterine contraction with myometrial extension or invasion.
